# Correction: Furugaito et al. Antimicrobial Susceptibility to 27 Drugs and the Molecular Mechanisms of Macrolide, Tetracycline, and Quinolone Resistance in *Gemella* sp. *Antibiotics* 2023, *12*, 1538

**DOI:** 10.3390/antibiotics13060515

**Published:** 2024-05-31

**Authors:** Michiko Furugaito, Yuko Arai, Yutaka Uzawa, Toshinori Kamisako, Kohei Ogura, Shigefumi Okamoto, Ken Kikuchi

**Affiliations:** 1 Department of Clinical Laboratory and Biomedical Sciences, Graduate School of Medicine, Osaka University, Suita, Osaka 565-0871, Japan; michiko-furugaito@med.kindai.ac.jp (M.F.); sokamoto@sahs.med.osaka-u.ac.jp (S.O.); 2 Department of Clinical Laboratory, Kindai University Hospital, Osakasayama, Osaka 589-8511, Japan; 3 Department of Infectious Diseases, Tokyo Women’s Medical University, Shinjuku-ku, Tokyo 162-8666, Japan; arai.yuko@twmu.ac.jp (Y.A.); uzawa.yutaka@twmu.ac.jp (Y.U.); 4 Department of Clinical Laboratory Medicine, Faculty of Medicine, Kindai University, Osakasayama, Osaka 589-8511, Japan; kamisako@med.kindai.ac.jp; 5 Division of Food Science and Biotechnology, Graduate School of Agriculture, Kyoto University, Uji, Kyoto 611-0011, Japan; ogura.kohei.7x@kyoto-u.ac.jp

## Text Correction

The authors wish to make the following corrections to this paper [1]. We request correction of the MICs and gene possession ratios of the two strains TWCC 52027 and TWCC 53044. This correction does not affect any content in our paper at all, but only affects a *p*-value described in Section 2.2.4 (Page 8): “whereas the ratio was higher in the GH group than in *G. morbillorum* (*p* = 0.16).” This *p*-value (0.16) was replaced with 0.08. We need to correct the values in Abstract, Results, Figures 1 and 2, Tables 2–5, Supplementary Table S1, Sections 2.2.2–2.2.4, Section 2.2.6, Sections 2.3–2.5.

**Abstract:** *Gemella* is a catalase-negative, facultative anaerobic, Gram-positive coccus that is commensal in humans but can become opportunistic and cause severe infectious diseases, such as infective endocarditis. Few studies have tested the antimicrobial susceptibility of *Gemella*. We tested its antimicrobial susceptibility to 27 drugs and defined the resistant genes using PCR in 58 *Gemella* strains, including 52 clinical isolates and 6 type strains. The type strains and clinical isolates comprised 22 *G. morbillorum*, 18 *G. haemolysans* (GH) group (genetically indistinguishable from *G. haemolysans* and *G. parahaemolysans*), 13 *G. taiwanensis*, three *G. sanguinis*, and two *G. bergeri*. No strain was resistant to beta-lactams and vancomycin. In total, 6/22 (27.3%) *G. morbillorum* strains were erythromycin- and clindamycin-resistant *ermB*-positive, whereas 5/18 (27.8%) in the GH group, 6/13 (46.2%) *G. taiwanensis*, and 1/3 (33.3%) of the *G. sanguinis* strains were erythromycin-non-susceptible *mefE*- or *mefA*-positive and clindamycin-susceptible. The MIC_90_ of minocycline and the ratios of *tetM*-positive strains varied across the different species—*G. morbillorum*: 2 µg/mL and 27.3% (6/22); GH group: 8 µg/mL and 22.2% (4/18); *G. taiwanensis*: 8 µg/mL and 53.8% (7/13), respectively. Levofloxacin resistance was significantly higher in *G. taiwanensis* (8/13, 61.5%) than in *G. morbillorum* (2/22, 9.1%). Levofloxacin resistance was associated with a substitution at serine 83 for leucine, phenylalanine, or tyrosine in GyrA. The mechanisms of resistance to erythromycin and clindamycin differed across *Gemella* species. In addition, the rate of susceptibility to levofloxacin differed across *Gemella* spp., and the quinolone resistance mechanism was caused by mutations in GyrA alone.

### *2.2.2.* *Susceptibility to Erythromycin*

In total, 20/58 strains were erythromycin-non-susceptible (intermediate or resistant), with MIC_90_ > 2 µg/mL. Although the ratios of the erythromycin-non-susceptible isolates varied across species, there was no significant difference among *G. morbillorum*, the GH group, and *G. taiwanensis*—*G. morbillorum*: 27.3% (6/22), GH group: 38.9% (7/18), *G. taiwanensis*: 46.2% (6/13), *G. sanguinis*: 33.3% (1/3), and *G. bergeri*: 0.0% (0/2) (Table 2, Figures 1 and 2).

### *2.2.3.* *Susceptibility to Clindamycin*

In total, 10/58 strains were clindamycin-non-susceptible, resulting in MIC_90_ > 2 µg/mL. Clindamycin-resistant *G. taiwanensis*, *G. sanguinis*, and *G. bergeri* isolates were not detected, and differences were not significant—*G. morbillorum*: 27.3% (6/22), GH group: 22.2% (4/18), *G. taiwanensis*: 0.0% (0/13), and *G. sanguinis*: 0.0% (0/3) (Table 2, Figures 1 and 2). Interestingly, all six erythromycin-resistant *G. morbillorum* strains were clindamycin-resistant. In contrast, 5/7 GH group strains, six strains of *G. taiwanensis*, and one *G. sanguinis* strain were erythromycin-non-susceptible and clindamycin-susceptible.

### *2.2.4.* *Susceptibility to Levofloxacin*

In total, 21/58 strains were levofloxacin-resistant, resulting in MIC_90_ > 128 µg/mL. Ratios of the levofloxacin strains varied across species—*G. morbillorum*: 9.1% (2/22), GH group: 50.0% (9/18), *G. taiwanensis*: 61.5% (8/13), *G. sanguinis*: 66.7% (2/3), and *G. bergeri*: 0.0% (0/2). The ratio of the resistant strains was significantly higher in *G. taiwanensis* than in *G. morbillorum* (*p* < 0.05 using chi-squared test), whereas the ratio was higher in the GH group than in *G. morbillorum* (*p* = 0.08) (Table 2, Figure 1).

### *2.2.6.* *Susceptibility to Other Antimicrobial Agents*

We tested the 18 antimicrobial agents whose breakpoints are not listed in CLSI M45-third edition. *Gemella* strains showed low MIC values for all beta-lactams: ampicillin, amoxicillin–clavulanic acid, sulbactam–ampicillin, cefazolin, cefdinir, cefepime, and imipenem (MIC_90_: ≤0.12, ≤0.25/0.12, ≤0.06/0.12, ≤0.25, ≤0.25, ≤0.06, and ≤0.06 μg/mL, respectively). The MIC_90_ values of clarithromycin and azithromycin were 8 and >4 µg/mL, respectively, consistent with those of erythromycin. The MIC_90_ values of clarithromycin varied among *G. morbillorum* (>16 μg/mL), the GH group (8 μg/mL), and *G. taiwanensis* (2 μg/mL), indicating the acquisition of high resistance to clarithromycin in *G. morbillorum* strains. The MIC_90_ value of moxifloxacin was high (>2 μg/mL) in *Gemella* strains. The MIC_90_ values of the aminoglycoside antibiotics gentamicin, gentamicin500 (to confirm tolerance to high concentrations of gentamicin), and arbekacin were 8, ≤500, and >8 μg/mL, respectively; sulfamethoxazole–trimethoprim, fosfomycin, and rifampicin were >38/2, ≤16, and ≤0.5 μg/mL, respectively; and the anti-MRSA agents teicoplanin, linezolid, and daptomycin were ≤0.5, 1, and 2 μg/mL, respectively (Table 2). Typically, streptococci are aminoglycoside-resistant. Therefore, we tested gentamicin500 to identify any *Gemella* strains that are highly resistant to aminoglycoside.

### 2.3. Phenotypes and Genotypes of Macrolide-Resistant Strains

The six erythromycin–clindamycin-resistant *G. morbillorum* strains exhibited constitutive resistance to macrolide, lincosamide, and streptogramin B (cMLSB). Their genotypes—*mefA/E*-negative, *ermB-*positive, and *msrA*-negative—were consistent with their phenotypes. Furthermore, five/seven strains of the GH group, six strains of *G. taiwanensis*, and one strain of *G. sanguinis* which were erythromycin-non-susceptible and clindamycin-susceptible, had macrolide-resistant (M) phenotypes and *mefE*- (four strains) or *mefA*-positive (one strain), *erm*-negative, and *msrA*-negative genotypes. In total, 2/7 GH group strains (TWCC 59567 and TWCC 59795) were erythromycin-resistant and clindamycin-non-susceptible and *mefE*-positive, but showed M phenotype. These results show that erythromycin-resistant *G. morbillorum* is associated with *ermB*, and erythromycin-non-susceptible GH-group, *G. taiwanensis*, and *G. sanguinis* are associated with *mefE*. The MIC values for clarithromycin were higher in the six *ermB*-positive *G. morbillorum* strains (8 or >16 µg/mL) (Table 3). All erythromycin-susceptible *Gemella* strains, except the *G. sanguinis* strain TWCC 70419, lacked *mefA/E*, *erm*, or *msrA* (Table S1).

### 2.4. Tetracycline Resistance

Next, we analyzed the possession rates of *tet*. Overall, 17/58 (29.3%) strains were *tetM*-positive; none of the other *tet* genes was detected. The ratios of *tetM*-positive strains in *G. morbillorum*, the GH group, *G. taiwanensis*, *G. sanguinis*, and *G. bergeri* were 27.3% (6/22), 33.3% (6/18), 38.5% (5/13), 0/3 (0.0%), and 0.0% (0/2), respectively. Among the 41 *tetM*-negative strains, one had minocycline (MIC = 2 µg/mL). The minocycline MIC values of the others were ≤1 µg/mL. The minocycline MIC of the 17 *tetM*-positive strains varied: ≤1 for five, 2 for five, and ≥8 µg/mL for seven strains, respectively (Table 4).

### 2.5. Mutations in gyrA and gyrB

We analyzed the *gyrA* and *gyrB* sequences. The 35 quinolone-susceptible strains possessed *gyrA*, encoding GyrA with a serine residue at 83 (S83). The serine residue was substituted with leucine (S83L), phenylalanine (S83F), or tyrosine (S83Y) in the 21 quinolone-resistant strains. Specifically, two *G. morbillorum* strains possessed GyrA/S83L, encoding *gyrA*. Seven of the GH group, seven *G. taiwanensis*, and two *G. sanguinis* strains contained S83F. Two in the GH group and one *G. taiwanensis* strains contained S84Y. GyrB mutations associated with levofloxacin resistance were not detected (Table 5).

**Figure 1:**
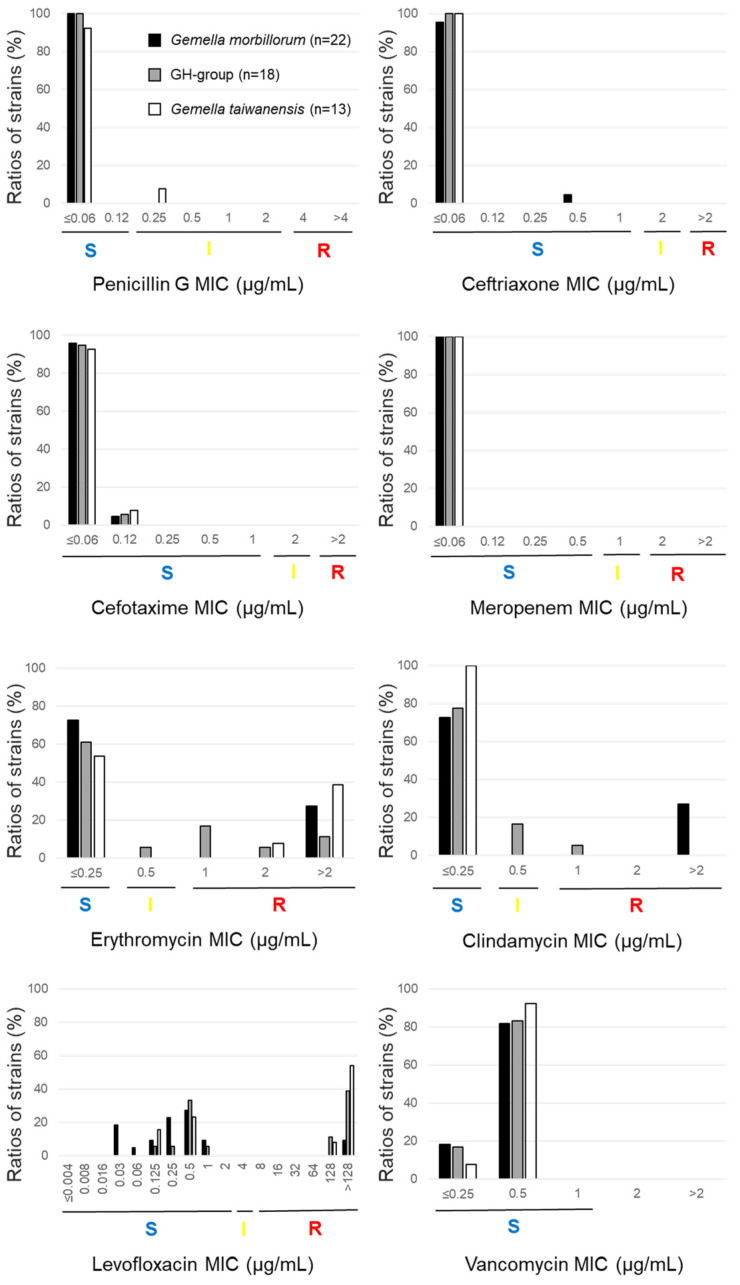
Ratios of resistant strains. S (blue), I (yellow), and R (red) indicate sensitive, intermediate, and resistant, respectively.

**Figure 2:**
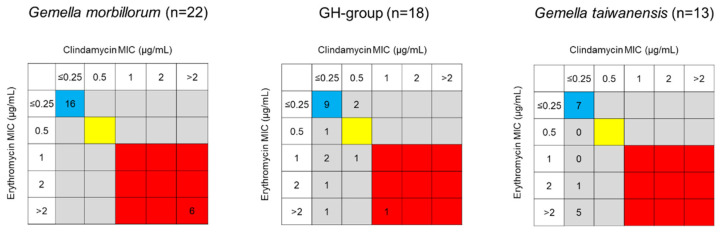
Distribution of erythromycin/clindamycin resistance in *Gemella* strains. Blue, yellow, and red boxes indicate sensitive, intermediate, and resistant, respectively.

**Table 2:** Susceptibility to antimicrobial agents with breakpoints listed in CLSI M45-third edition.

Antimicrobial Agents/ *Gemella* spp.	MIC (μg/mL)			Interpretive Breakpoint (μg/mL) ^a^ or % of Isolates		
	Range	MIC_50_	MIC_90_	Susceptible	Intermediate	Resistant
Penicillin G ^c^	≤0.06–>4			≤0.12	0.25–2	≥4
*Gemella morbillorum*	≤0.06	≤0.06	≤0.06	100.0	0.0	0.0
GH group	≤0.06	≤0.06	≤0.06	100.0	0.0	0.0
*Gemella taiwanensis*	≤0.06–0.25	≤0.06	≤0.06	92.3	7.7	0.0
*Gemella sanguinis*	≤0.06	–	–	100.0	0.0	0.0
*Gemella bergeri*	≤0.06	–	–	100.0	0.0	0.0
Total	≤0.06–0.25	≤0.06	≤0.06	98.3	1.7	0.0
Ampicillin	≤0.12–>4					
*Gemella morbillorum*	≤0.12–0.25	≤0.12	≤0.12	NA ^b^	NA	NA
GH group	≤0.12	≤0.12	≤0.12	NA	NA	NA
*Gemella taiwanensis*	≤0.12–0.5	≤0.12	≤0.12	NA	NA	NA
*Gemella sanguinis*	≤0.12	–	–	NA	NA	NA
*Gemella bergeri*	≤0.12	–	–	NA	NA	NA
Total	≤0.12–0.5	≤0.12	≤0.12	NA	NA	NA
Amoxicillin–clavulanic acid	≤0.25/0.12–>4/2					
*Gemella morbillorum*	≤0.25/0.12	≤0.25/0.12	≤0.25/0.12	NA	NA	NA
GH group	≤0.25/0.12	≤0.25/0.12	≤0.25/0.12	NA	NA	NA
*Gemella taiwanensis*	≤0.25/0.12	≤0.25/0.12	≤0.25/0.12	NA	NA	NA
*Gemella sanguinis*	≤0.25/0.12	–	–	NA	NA	NA
*Gemella bergeri*	≤0.25/0.12	–	–	NA	NA	NA
Total	≤0.25/0.12	≤0.25/0.12	≤0.25/0.12	NA	NA	NA
Sulbactam–ampicillin	≤0.06/0.12–>2/4					
*Gemella morbillorum*	≤0.06/0.12	≤0.06/0.12	≤0.06/0.12	NA	NA	NA
GH group	≤0.06/0.12	≤0.06/0.12	≤0.06/0.12	NA	NA	NA
*Gemella taiwanensis*	≤0.06/0.12–0.25/0.5	≤0.06/0.12	≤0.06/0.12	NA	NA	NA
*Gemella sanguinis*	≤0.06/0.12	–	–	NA	NA	NA
*Gemella bergeri*	≤0.06/0.12	–	–	NA	NA	NA
Total	≤0.06/0.12–0.25/0.5	≤0.06/0.12	≤0.06/0.12	NA	NA	NA
Cefazolin	≤0.25–>2					
*Gemella morbillorum*	≤0.25	≤0.25	≤0.25	NA	NA	NA
GH group	≤0.25–0.5	≤0.25	0.5	NA	NA	NA
*G* *emellataiwanensis*	≤0.25–0.5	≤0.25	0.5	NA	NA	NA
*Gemella sanguinis*	≤0.25	–	–	NA	NA	NA
*Gemella bergeri*	≤0.25	–	–	NA	NA	NA
Total	≤0.25–0.5	≤0.25	≤0.25	NA	NA	NA
Cefdinir	≤0.25–>1					
*G* *emella morbillorum*	≤0.25	≤0.25	≤0.25	NA	NA	NA
GH group	≤0.25	≤0.25	≤0.25	NA	NA	NA
*Gemella taiwanensis*	≤0.25	≤0.25	≤0.25	NA	NA	NA
*Gememlla sanguinis*	≤0.25–0.5	–	–	NA	NA	NA
*Gemella bergeri*	≤0.25	–	–	NA	NA	NA
Total	≤0.25–0.5	≤0.25	≤0.25	NA	NA	NA
Ceftriaxone ^c^	≤0.06–>2			≤1	2	≥4
*Gemella morbillorum*	≤0.06–0.5	≤0.06	≤0.06	100.0	0.0	0.0
GH group	≤0.06	≤0.06	≤0.06	100.0	0.0	0.0
*Gemella taiwanensis*	≤0.06	≤0.06	≤0.06	100.0	0.0	0.0
*Gemella sanguinis*	0.25–1	–	–	100.0	0.0	0.0
*Gemella bergeri*	≤0.06			100.0	0.0	0.0
Total	≤0.06–1	≤0.06	≤0.06	100.0	0.0	0.0
Cefotaxime ^c^	≤0.06–>2			≤1	2	≥4
*Gemella morbillorum*	≤0.06–0.12	≤0.06	≤0.06	100.0	0.0	0.0
GH group	≤0.06–0.12	≤0.06	≤0.06	100.0	0.0	0.0
*Gemella taiwanensis*	≤0.06–0.12	≤0.06	≤0.06	100.0	0.0	0.0
*Gemella sanguinis*	0.25–1	–	–	100.0	0.0	0.0
*Gemella bergeri*	≤0.06	–	–	100.0	0.0	0.0
Total	≤0.06–1	≤0.06	0.12	100.0	0.0	0.0
Cefepime	≤0.06–>2					
*G* *emella morbillorum*	≤0.06–0.5	≤0.06	≤0.06	NA	NA	NA
GH group	≤0.06–0.12	≤0.06	0.12	NA	NA	NA
*Gemella taiwanensis*	≤0.06–0.12	≤0.06	≤0.06	NA	NA	NA
*Gemella sanguinis*	0.25–1	–	–	NA	NA	NA
*Gemella bergeri*	≤0.06	–	–	NA	NA	NA
Total	≤0.06–1	≤0.06	0.12	NA	NA	NA
Imipenem	≤0.06–>4					
*Gemella morbillorum*	≤0.06	≤0.06	≤0.06	NA	NA	NA
GH group	≤0.06	≤0.06	≤0.06	NA	NA	NA
*Gemella taiwanensis*	≤0.06	≤0.06	≤0.06	NA	NA	NA
*Gemella sanguinis*	≤0.06	–	–	NA	NA	NA
*Gemella bergeri*	≤0.06	–	–	NA	NA	NA
Total	≤0.06	≤0.06	≤0.06	NA	NA	NA
Meropenem ^c^	≤0.06–>2			≤0.5	1	≥2
*Gemella morbillorum*	≤0.06	≤0.06	≤0.06	100.0	0.0	0.0
GH group	≤0.06	≤0.06	≤0.06	100.0	0.0	0.0
*Gemella taiwanensis*	≤0.06	≤0.06	≤0.06	100.0	0.0	0.0
*Gemella sanguinis*	≤0.06	–	–	100.0	0.0	0.0
*Gemella bergeri*	≤0.06	–	–	100.0	0.0	0.0
Total	≤0.06	≤0.06	≤0.06	100.0	0.0	0.0
Erythromycin ^c^	≤0.25–>2			≤0.25	0.5	≥1
*Gemella morbillorum*	≤0.25–>2	≤0.25	>2	72.7	0.0	27.3
GH group	≤0.25–>2	≤0.25	>2	61.1	5.6	33.3
*Gemella taiwanensis*	≤0.25–>2	≤0.25	>2	53.8	0.0	46.2
*Gemella sanguinis*	≤0.25–1	–	–	66.7	0.0	33.3
*Gemella bergeri*	≤0.25	–	–	100.0	0.0	0.0
Total	≤0.25–>2	≤0.25	>2	65.5	1.7	32.8
Clarithromycin	≤0.12–>16					
*Gemella morbillorum*	≤0.12–>16	≤0.12	>16	NA	NA	NA
GH group	≤0.12–16	≤0.12	8	NA	NA	NA
*Gemella taiwanensis*	≤0.12–8	≤0.12	2	NA	NA	NA
*Gemella sanguinis*	≤0.12–0.25	–	–	NA	NA	NA
*Gemella bergeri*	≤0.12	–	–	NA	NA	NA
Total	≤0.12–>16	≤0.12	8	NA	NA	NA
Azithromycin	≤0.12–>4					
*Gemella morbillorum*	≤0.12–>4	≤0.12	>4	NA	NA	NA
GH group	≤0.12–>4	≤0.12	>4	NA	NA	NA
*Gemella taiwanensis*	≤0.12–>4	0.25	>4	NA	NA	NA
*Gemella sanguinis*	0.25–4	–	–	NA	NA	NA
*Gemella bergeri*	0.25	–	–	NA	NA	NA
Total	≤0.12–>4	≤0.12	>4	NA	NA	NA
Clindamycin ^c^	≤0.25–>2			≤0.25	0.5	≥1
*Gemella morbillorum*	≤0.25–>2	≤0.25	>2	72.7	0.0	27.3
GH group	≤0.25–1	≤0.25	0.5	77.8	16.7	5.6
*Gemella taiwanensis*	≤0.25	≤0.25	≤0.25	100.0	0.0	0.0
*Gemella sanguinis*	≤0.25	–	–	100.0	0.0	0.0
*Gemella bergeri*	≤0.25	–	–	100.0	0.0	0.0
Total	≤0.25–>2	≤0.25	>2	82.8	5.2	12.1
Erythromycin/clindamycin	≤1/0.5–>1/0.5					
*G. morbillorum*	≤1/0.5–>1/0.5	≤1/0.5	>1/0.5	NA	NA	NA
GH group	≤1/0.5–>1/0.5	≤1/0.5	≤1/0.5	NA	NA	NA
*G. taiwanensis*	≤1/0.5	≤1/0.5	≤1/0.5	NA	NA	NA
*G. sanguinis*	≤1/0.5	–	–	NA	NA	NA
*Gemella bergeri*	≤1/0.5	–	–	NA	NA	NA
Total	≤1/0.5–>1/0.5	≤1/0.5	>1/0.5	NA	NA	NA
Levofloxacin ^c^	≤0.004–>128			≤2	4	≥8
*Gemella morbillorum*	0.03–>128	0.25	1	90.9	0.0	9.1
GH group	0.125–>128	1	>128	50.0	0.0	50.0
*Gemella taiwanensis*	0.125–>128	>128	>128	38.5	0.0	61.5
*Gemella sanguinis*	0.5–>128	–	–	33.3	0.0	66.7
*Gemella bergeri*	0.5	–	–	100.0	0.0	0.0
Total	0.03–>128	0.5	>128	63.8	0.0	36.2
Moxifloxacin	≤0.5–>2					
*G* *emella morbillorum*	≤0.5–>2	≤0.5	>2	NA	NA	NA
GH group	≤0.5–>2	≤0.5	>2	NA	NA	NA
*Gemella taiwanensis*	≤0.5–>2	>2	>2	NA	NA	NA
*Gemella sanguinis*	≤0.5–>2	–	–	NA	NA	NA
*Gemella bergeri*	≤0.5	–	–	NA	NA	NA
Total	≤0.5–>2	≤0.5	>2	NA	NA	NA
Minocycline	≤1–>8					
*Gemella morbillorum*	≤1–>8	≤1	2	NA	NA	NA
GH group	≤1–8	≤1	8	NA	NA	NA
*Gemella taiwanensis*	≤1–8	≤1	8	NA	NA	NA
*Gemella sanguinis*	≤1	–	–	NA	NA	NA
*Gemella bergeri*	≤1	–	–	NA	NA	NA
Total	≤1	≤1	8	NA	NA	NA
Sulfamethoxazole– trimethoprim	≤9.5/0.5–>38/2					
*Gemella morbillorum*	≤9.5/0.5–>38/2	19/1	>38/2	NA	NA	NA
GH group	≤9.5/0.5–>38/2	38/2	>38/2	NA	NA	NA
*Gemella taiwanensis*	≤9.5/0.5–>38/2	19/1	19/1	NA	NA	NA
*Gemella sanguinis*	19/1–>38/2	–	–	NA	NA	NA
*Gemella bergeri*	≤9.5/0.5	–	–	NA	NA	NA
Total	≤9.5/0.5–>38/2	19/1	>38/2	NA	NA	NA
Gentamicin	≤1–>8					
*Gemella morbillorum*	≤1–8	2	8	NA	NA	NA
GH group	≤1–2	≤1	2	NA	NA	NA
*Gemella taiwanensis*	≤1–4	2	4	NA	NA	NA
*Gemella sanguinis*	≤1–8	–	–	NA	NA	NA
*Gemella bergeri*	2, 4	–	–	NA	NA	NA
Total	≤1–8	2	8	NA	NA	NA
Gentamicin 500	≤500–>500					
*Gemella morbillorum*	≤500	≤500	≤500	NA	NA	NA
GH group	≤500	≤500	≤500	NA	NA	NA
*Gemella taiwanensis*	≤500	≤500	≤500	NA	NA	NA
*Gemella sanguinis*	≤500	–	–	NA	NA	NA
*Gemella bergeri*	≤500	–	–	NA	NA	NA
Total	≤500	≤500	≤500	NA	NA	NA
Arbekacin	≤1–>8					
*Gemella morbillorum*	≤1–8	8	>8	NA	NA	NA
GH group	≤1–8	4	8	NA	NA	NA
*Gemella taiwanensis*	2–>8	4	8	NA	NA	NA
*Gemella sanguinis*	4–>8	–	–	NA	NA	NA
*Gemella bergeri*	4, >8	–	–	NA	NA	NA
Total	≤1–8	4	>8	NA	NA	NA
Fosfomycin	≤16–>128					
*G* *emella morbillorum*	≤16–32	≤16	≤16	NA	NA	NA
GH group	≤16	≤16	≤16	NA	NA	NA
*Gemella taiwanensis*	≤16	≤16	≤16	NA	NA	NA
*Gemalla sanguinis*	≤16	–	–	NA	NA	NA
*Gemella bergeri*	≤16	–	–	NA	NA	NA
Total	≤16–32	≤16	≤16	NA	NA	NA
Rifampicin	≤0.5–>2					
*Gemella morbillorum*	≤0.5	≤0.5	≤0.5	NA	NA	NA
GH group	≤0.5	≤0.5	≤0.5	NA	NA	NA
*Gemella taiwanensis*	≤0.5	≤0.5	≤0.5	NA	NA	NA
*Gemella sanguinis*	≤0.5	–	–	NA	NA	NA
*Gemella bergeri*	≤0.5			NA	NA	NA
Total	≤0.5	≤0.5	≤0.5	NA	NA	NA
Vancomycin ^c^	≤0.25–>2			≤1		
*Gemella morbillorum*	≤0.25–0.5	0.5	0.5	100.0	0.0	0.0
GH group	≤0.25–0.5	0.5	0.5	100.0	0.0	0.0
*Gemella taiwanensis*	≤0.25–0.5	0.5	0.5	100.0	0.0	0.0
*Gemella sanguinis*	≤0.25–0.5	–	–	100.0	0.0	0.0
*Gemella bergeri*	0.5	–	–	100.0	0.0	0.0
Total	≤0.25–0.5	0.5	0.5	100.0	0.0	0.0
Teicoplanin	≤0.5–>16					
*Gemella morbillorum*	≤0.5	≤0.5	≤0.5	NA	NA	NA
GH group	≤0.5	≤0.5	≤0.5	NA	NA	NA
*Gemella taiwanensis*	≤0.5	≤0.5	≤0.5	NA	NA	NA
*Gemella sanguinis*	≤0.5	–	–	NA	NA	NA
*Gemella bergeri*	≤0.5	–	–	NA	NA	NA
Total	≤0.5	≤0.5	≤0.5	NA	NA	NA
Linezolid	≤0.5–>4					
*Gemella morbillorum*	≤0.5–1	≤0.5	1	NA	NA	NA
GH group	≤0.5–1	≤0.5	1	NA	NA	NA
*Gemella taiwanensis*	≤0.5	≤0.5	≤0.5	NA	NA	NA
*Gemella sanguinis*	≤0.5–1	–	–	NA	NA	NA
*Gemella bergeri*	≤0.5, 2	–	–	NA	NA	NA
Total	≤0.5	≤0.5	1	NA	NA	NA
Daptomycin	≤0.25–>4					
*Gemella morbillorum*	≤0.25–4	2	2	NA	NA	NA
GH group	0.5–2	1	2	NA	NA	NA
*Gemella taiwanensis*	≤0.25–2	1	2	NA	NA	NA
*Gemella sanguinis*	1–4	–	–	NA	NA	NA
*Gemella bergeri*	2, 4	–	–	NA	NA	NA
Total	≤0.25–4	1	2	NA	NA	NA

^a^ Interpretive breakpoints are shown in bold for each antibiotic. ^b^ NA, not applicable (breakpoints not established). ^c^ Antimicrobial agents with breakpoints listed in CLSI M45-third edition.

**Table 3:** Distribution of macrolides and clindamycin MICs and possession of the *mef*, *erm,* and *msrA* genes in erythromycin-non-susceptible *Gemella* isolates.

Strain No.	Identification	MIC (μg/mL)					Macrolide Phenotype ^a,b^	*mefA/E*	*erm*	*msrA*
		Erythromycin	Clindamycin	Erythromycin/Clindamycin	Clarithromycin	Azithromycin				
TWCC 57201	*Gemella morbillorum*	>2	>2	>1/0.5	8	>4	cMLS_B_	-	*ermB*	*-*
TWCC 57818	*Gemella morbillorum*	>2	>2	>1/0.5	>16	>4	cMLS_B_	-	*ermB*	*-*
TWCC 57944	*Gemella morbillorum*	>2	>2	>1/0.5	>16	>4	cMLS_B_	-	*ermB*	*-*
TWCC 59111	*Gemella morbillorum*	>2	>2	>1/0.5	8	>4	cMLS_B_	-	*ermB*	*-*
TWCC 71703	*Gemella morbillorum*	>2	>2	>1/0.5	>16	>4	cMLS_B_	-	*ermB*	*-*
TWCC 72266	*Gemella morbillorum*	>2	>2	>1/0.5	>16	>4	cMLS_B_	-	*ermB*	*-*
TWCC 52027	GH group	0.5	≤0.25	≤1/0.5	8	2	M	*mefE*	-	-
TWCC 59566	GH group	2	≤0.25	≤1/0.5	2	>4	M	*mefE*	-	-
TWCC 59567	GH group	>2	1	>1/0.5	16	>4	M	*mefE*	-	-
TWCC 59795	GH group	1	0.5	≤1/0.5	0.5	2	M	*mefE*	-	-
TWCC 70939	GH group	>2	≤0.25	≤1/0.5	2	>4	M	*mefE*	-	-
TWCC 71200	GH group	1	≤0.25	≤1/0.5	2	2	M	*mefA*	-	-
TWCC 71814	GH group	1	≤0.25	≤1/0.5	0.5	1	M	*mefE*	-	-
TWCC 55344	*Gemella taiwanensis*	>2	≤0.25	≤1/0.5	8	>4	M	*mefE*	-	-
TWCC 58522	*Gemella taiwanensis*	>2	≤0.25	≤1/0.5	2	4	M	*mefE*	-	-
TWCC 70386	*Gemella taiwanensis*	>2	≤0.25	≤1/0.5	2	4	M	*mefE*	-	-
TWCC 72085	*Gemella taiwanensis*	>2	≤0.25	≤1/0.5	2	>4	M	*mefE*	-	-
TWCC 70387L	*Gemella taiwanensis*	2	≤0.25	≤1/0.5	0.5	>4	M	*mefE*	-	-
TWCC 70387S	*Gemella taiwanensis*	>2	≤0.25	≤1/0.5	2	>4	M	*mefE*	-	-
TWCC 54965	*Gemella sanguinis*	1	≤0.25	≤1/0.5	0.25	4	M	*mefE*	-	-
TWCC 70419	*Gemella sanguinis*	≤0.25 ^c^	≤0.25	≤1/0.5	≤0.12	0.25	not M	*mefE*	-	-

^a^ cMLSB: macrolide–lincosamide–streptogramin B-resistant phenotype. ^b^ M: macrolide-resistant phenotype. ^c^ Erythromycin-susceptible.

**Table 4:** Distribution of minocycline MIC and *ermB* in *Gemella* isolates harboring the *tetM* gene.

Strain No.	Identification	*tetM*	Minocycline MIC (μg/mL)	*ermB*
TWCC 57944	*Gemella morbillorum*	+	2	+
TWCC 57987	*Gemella morbillorum*	+	≤1	−
TWCC 59111	*Gemella morbillorum*	+	2	+
TWCC 70937	*Gemella morbillorum*	+	>8	−
TWCC 71703	*Gemella morbillorum*	+	2	+
TWCC 72266	*Gemella morbillorum*	+	≤1	+
TWCC 51800	GH group	+	8	−
TWCC 59795	GH group	+	≤1	−
TWCC 70939	GH group	+	8	−
TWCC 71814	GH group	+	2	−
TWCC 53044	*Gemella taiwanensis*	+	8	−
TWCC 56546	*Gemella taiwanensis*	+	2	−
TWCC 58522	*Gemella taiwanensis*	+	8	−
TWCC 70386	*Gemella taiwanensis*	+	8	−
TWCC 72085	*Gemella taiwanensis*	+	8	−
TWCC 70387L	*Gemella taiwanensis*	+	≤1	−
TWCC 70387S	*Gemella taiwanensis*	+	≤1	−

**Table 5:** Distribution of MIC of tested quinolones and amino acid substitutions in *gyrA* gene in quinolone-resistant *Gemella* isolates.

Strain	*n*	MIC (μg/mL)		GyrA Amino Acid Substitutions ^a^
		Levofloxacin	Moxifloxacin	
*Gemella morbillorum*	2	>128	>2	Ser83 > Leu83 (*n* = 2)
GH group	9	128–>128	>2	Ser83 > Phe83 (*n* = 7), Ser83 > Tyr83 (*n* =2)
*Gemella taiwanensis*	8	128–>128	>2	Ser83 > Phe83 (*n* = 7), Ser83 > Tyr83 (*n* = 1)
*Gemella sanguinis*	2	128–>128	>2	Ser83 > Phe83 (*n* = 2)

^a^ *gyrA*-Ser83 Leu: serine to leucine at codon 83; Ser83 Phe: serine to phenylalanine at codon 83; Ser83 Tyr; serine to tyrosine at codon 83.

The authors state that the scientific conclusions are unaffected. This correction was approved by the Academic Editor. The original publication has also been updated.
